# A patient with Graves’ disease showing only psychiatric symptoms and negativity for both TSH receptor autoantibody and thyroid stimulating antibody

**DOI:** 10.1186/1756-6614-5-19

**Published:** 2012-12-03

**Authors:** Hidetaka Hamasaki, Taro Yoshimi, Hidekatsu Yanai

**Affiliations:** 1 Department of Internal Medicine, National Center for Global Health and Medicine Kohnodai Hospital, Chiba, Japan; 2 Department of Psychiatry, National Center for Global Health and Medicine Kohnodai Hospital, Chiba, Japan

**Keywords:** Delusion, Hyperthyroidism, Scintigraphy, Thyroid stimulating autoantibody, TSH receptor autoantibody

## Abstract

**Background:**

Both thyroid stimulating hormone (TSH) and thyroid stimulating antibody (TSAb) negative Graves’s disease (GD) is extremely rare. Here we present such a patient.

**Case presentation:**

The patient was a 76-year-old woman who was diagnosed as having schizophrenia forty years ago. She did not show characteristic symptoms for hyperthyroidism, such as swelling of thyroid, exophthalmos, tachycardia and tremor, however, she showed only psychomotor agitation. Serum free triiodothyronine and free thyroxine levels were elevated and TSH level was suppressed, suggesting the existence of hyperthyroidism. However, both the first generation TSH receptor autoantibody (TRAb1) and the thyroid stimulating autoantibody (TSAb) were negative. Slightly increased blood flow and swelling was detected by thyroid echography. Thyroid scintigraphy demonstrated diffuse and remarkably elevated uptake of ^123^I uptake. Finally, we diagnosed her as having GD. She was treated by using methimazole, and hyperthyroidism and her psychiatric symptoms were promptly ameliorated.

**Discussion:**

We experienced a patient with GD who did not show characteristic symptoms except for psychiatric symptoms, and also showed negativity for both TRAb1 and TSAb. Thyroid autoantibody-negative GD is extremely rare. Thyroid scintigraphy was useful to diagnose such a patient.

## Introduction

Graves’ disease (GD) is induced by autoimmunity for the thyroid gland, and thyroid stimulating autoantibodies may be the main pathological cause of GD. We usually measure thyroid stimulating hormone (TSH) receptor autoantibodies for diagnosis for GD in medical practices. In fact, TSH receptor autoantibodies are detectable in over 90% of patients with GD [1].

Here we present a patient with GD showing the absence of characteristic symptoms and negativity for both the first generation TSH receptor autoantibody (TRAb1) and thyroid stimulating antibody (TSAb).

### Case report

A 76–year-old woman was admitted to the Department of Psychiatry in our hospital because she denied medications and suffered from irritability and delusion. She had no complaints of somatic symptoms. Her thyroid function was normal at least four months before the admission, however, laboratory data on the admission revealed hyperthyroidism. She was pointed out to have thyroid disease thirty years ago, however, she was not diagnosed definitively at that time, and also did not take any medicine. She had also type 2 diabetes, hypertension, dyslipidemia, hyperuricemia and schizophrenia. The members of her family did not have thyroid disorders. She has been taking clonazepam (0.5 mg/day), trazodone (50 mg/day), nitrazepam (5 mg/day), 0.1% aripiprazole (6 ml/day), propranolol (30 mg/day) and telmisartan (40 mg/day), for the treatment for hypertension, psychiatric symptoms and schizophrenia.

**Figure 1:**
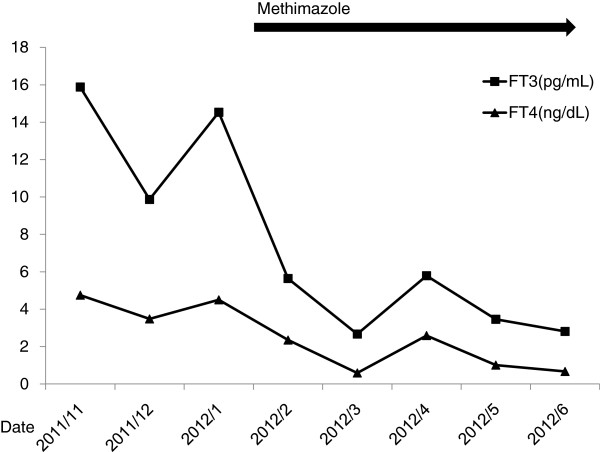
Changes of free triiodothyronine (FT3) and free thyroxine (FT4).

Her body temperature was 35.7°C, blood pressure was 158/86 mmHg, and heart rate was 89 per minute and regular. Her thyroid was not swelled. She did not have tremor and exophthalmos. The serum TSH level was suppressed to < 0.03 μIU/mL (normal: 0.54-4.26 μIU/mL), free triiodothyronine (T3) and free thyroxine (T4) levels were elevated to 11.70 pg/mL (normal: 2.39-4.06 pg/mL) and 3.07 ng/dL (normal: 0.71-1.52 ng/dL), respectively (Figure 1). Plasma glucose level was 116 mg/dL (normal: 80–112 mg/dL), and HbA1c level was 6.6% (normal: 4.7-6.2%). Her liver and renal functions were normal, and leukocyte counts and C-reactive protein (CRP) level were not elevated.

**Figure 2:**
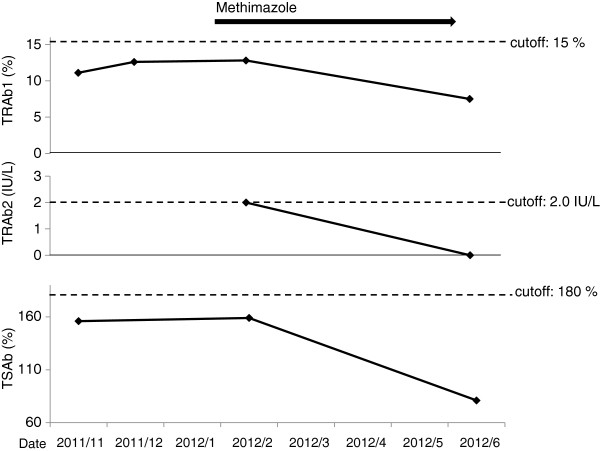
**Changes of the first generation TSH receptor autoantibody (TRAb1), the second generation TSH receptor autoantibody (TRAb2) and thyroid stimulating antibody (TSAb).** The cutoff points for TRAb1 and TSAb are values recommended by manufactures. The cutoff point for TRAb2 is value recommended by Schott M, et al. [10].

**Figure 3:**
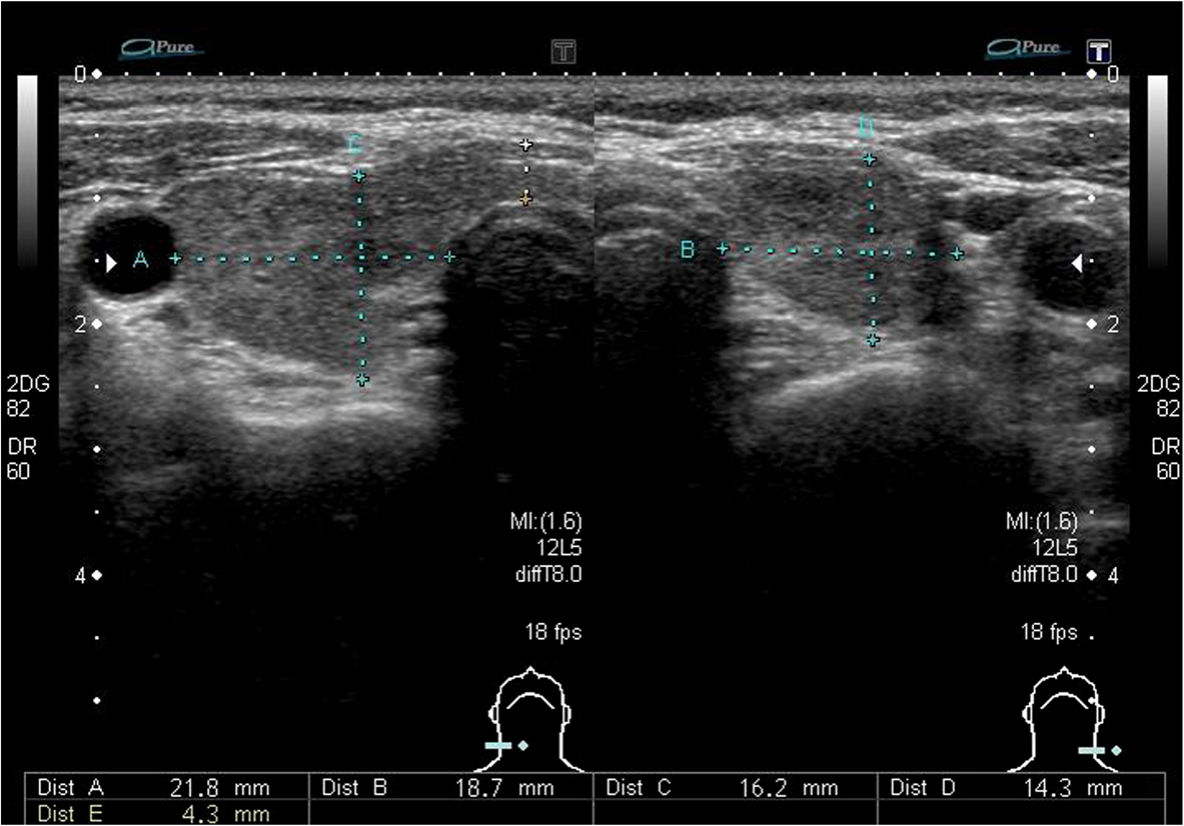
Echography of the thyroid glands. Bilateral thyroid glands was slightly swelled.

**Figure 4:**
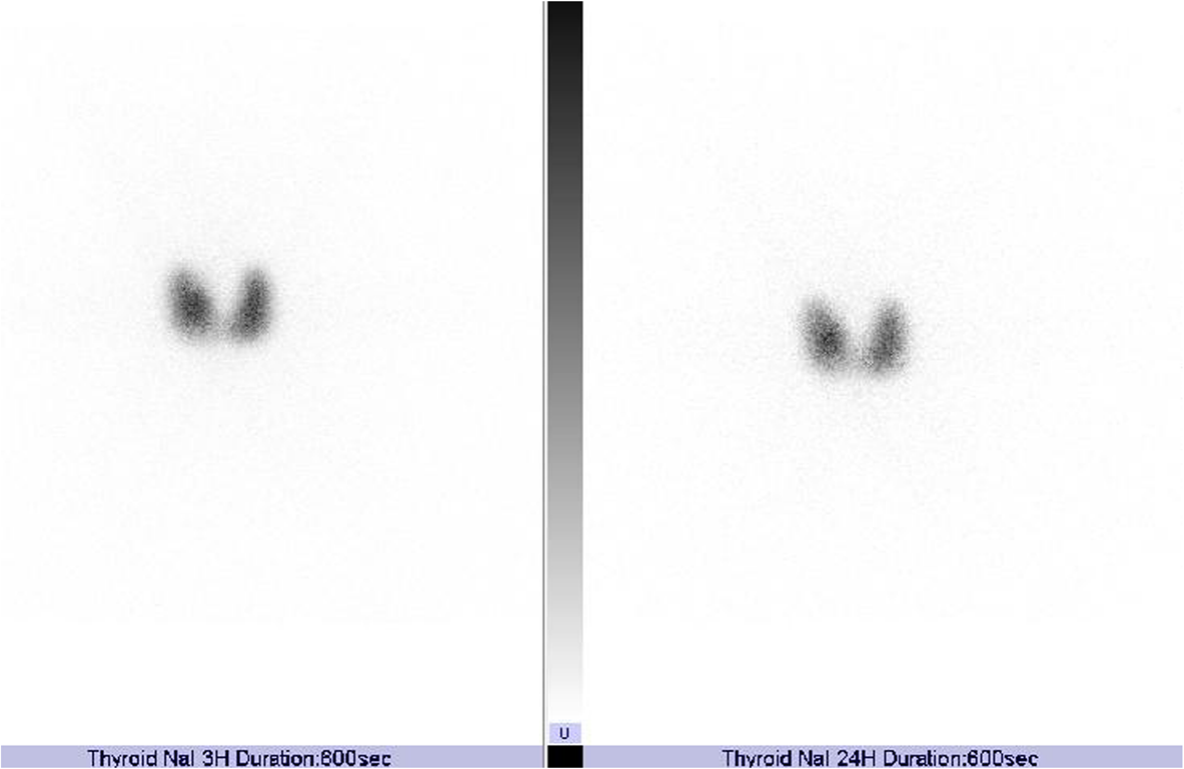
**Scintigraphy of the thyroid glands.** Uptake rate 3 and 24 hours after injection of ^123^I was 111% and 63% respectively.

Her serum levels of TRAb1 (7.7%; the cutoff point: 15%) and TSAb (155%; the cutoff point: 180%) were not elevated (Figure 2). Serum anti-thyroid peroxidase (TPO) antibody was elevated to 174 IU/mL (normal: < 16 IU/mL). At first, we diagnosed her as having painless thyroiditis, and did not give her an anti-thyroid drug. Her psychiatric symptoms continued and we re-evaluated her thyroid function one month later. The serum TSH level was still suppressed to < 0.03 μIU/mL (normal: 0.54-4.26 μIU/mL), and free T3 and free T4 levels were further elevated to 15.88 pg/mL and 4.75 ng/dL, respectively (Figure 1). Thyroid echography showed slight swelling of the thyroid gland (right lobe: 52.4 mm × 21.8 mm × 16.4 mm, left lobe: 46.6 mm × 18.7 mm × 12.8 mm, isthmus size: 4.3 mm) and slightly increased blood flow (Figure 3). Thyroid scintigraphy demonstrated a diffuse and significantly elevated uptake of ^123^I uptake in her thyroid gland (Figure 4). Uptake rate 3 and 24 hours after injection of ^123^I was 111% and 63%, respectively Her serum levels of TRAb1 and TSAb were still not elevated (Figure 2), in spite of elevation of anti-TPO antibody (210 IU/mL; normal: < 16 IU/mL). We evaluated second generation TRAb (TRAb2), and found that her serum level of TRAb2 was very weakly positive (2.0 IU/mL; the cutoff point: 1.0 IU/mL). Because results of thyroid echography and scintigraphy strongly suggested the development of GD, we treated her by using methimazole (30 mg/day). Her thyroid function was promptly normalized one month later (Figure 1), and her psychiatric symptoms also were ameliorated. Further, the values of TRAb1, TRAb2 and TSAb were also promptly decreased under each cutoff point (Figure 2).

## Discussion

Hyperthyroidism is a condition where the thyroid gland abnormally produces too much thyroid hormones. The most common cause is GD [2,3], and which is developed by the autoimmune stimulation of the thyroid gland [1]. Standardized diagnostic criteria for GD has not been globally established, and we usually diagnose this disease by clinical manifestations, laboratory data and imaging studies [4]. Clinical symptoms and signs include body weight loss, tremor, irritability, tachycardia, goiter, exophthalmos. Our patient had no specific somatic symptom of GD. Adrenergic hyperactivity induces psychiatric symptoms in hyperthyroidism, however. GD patients presenting only delusion like our patient are uncommon [5,6].

Paunkovic analyzed 255 patients diagnosed as having GD based on clinical manifestations and laboratory findings (free T4 and TSH). They excluded TRAb positive patients and the rest of the patients who had hyperthyroidism were re-tested with thyroid scintigraphy and serum levels of another TRAb. These results showed that TRAb were detectable in 98.7% of patients with GD. They stated TRAb negative GD was extremely rare, but there were methodological limitations in the assessment of only TRAb for the valid diagnosis of GD [7].

Our case showed no evident clinical manifestations except for psychomotor agitation which was considered to be induced by schizophrenia, and also showed negativity for both TRAb1 and TSAb. We observed a very weak positivity of TRAb2 before we started treatment. However, TRAb or TSAb have been reported to emerge in about 10% of patients with painless thyroiditis [8,9], therefore, we cannot make a definite differential diagnosis between GD and painless thyroiditis even if one of the thyroid autoantibodies is very weakly positive. The recommended cut-off value of TRAb2 used to be 2.0 IU/L and the sensitivity is 93.9% with 100% specificity [10]. It may be evident that the autoimmune stimulation of the thyroid gland is the cause of hyperthyroidism in our patient because serum levels of thyroid autoantibodies (TRAb1, TRAb2 and TSAb) were significantly decreased after starting treatment (Figure 2). We believe that present case is very rare. Our study also suggested that thyroid scintigraphy is very useful to diagnose GD patients with the absence of characteristic symptoms, TRAb and TSAb.

Thyroid autoantibody-negative GD is extremely rare. We experienced a patient with GD who did not show characteristic symptoms except for psychiatric symptoms, and also showed negativity for both TRAb1 and TSAb. Thyroid scintigraphy was useful to diagnose such a patient.

### Consent

Written informed consent was obtained from the patient's relatives for publication of this case report. A copy of the written consent is available for review by the Eitor-in-Chief of this jounal.

## Competing interests

The authors declare that they have no competing interests.

## Authors’ contributions

HH wrote the first draft. HH and TY were in a position of leadership for the patient and collected information on the patient. HY and HH did the literature searches. HY wrote the final manuscript and made appropriate revisions. All authors read through and approved the final manuscript.
